# 570. Prioritized Access to COVID-19 Vaccines Among Vulnerable Communities Increases Vaccination Rates

**DOI:** 10.1093/ofid/ofab466.768

**Published:** 2021-12-04

**Authors:** Leonor Fernandez, Ashley O'Donoghue, Peter Shorett, Jonathan Blair, Lawrence Markson, Tenzin Dechen, Jennifer Stevens, Sharon Wright

**Affiliations:** 1 Beth Israel Deaconess Medical Center, Boston, MA; 2 Beth Israel Lahey Health, Boston, MA

## Abstract

**Background:**

Based on national recommendations,^1^ Beth Israel Lahey Health (BILH) in Eastern Massachusetts (MA) prioritized vulnerable communities in our distribution of COVID-19 vaccines. We hypothesized that creating prioritized access to appointments for patients in these communities would increase the likelihood vaccination.

**Methods:**

The BILH health system sent vaccine invitations first to patients of two clinics in vulnerable neighborhoods in Boston (Wave 1), followed by other patients from vulnerable communities (Wave 2) up to 1 day later, and then by all other patients (Wave 3) after up to 1 more day later. To identify whether early access/prioritization increased the likelihood of receipt of vaccine at any site or a vaccine at a BILH clinic, we compared patients in Wave 1 in a single community with high cumulative incidence of COVID-19 (Dorchester) to patients in Wave 2 during a period of limited vaccine access, 1/27/21-2/24/21. Each wave was modeled using logistic regression, adjusted for language and race. By taking the difference between these two differences, we are left with the impact of early vaccination invitation in Wave 1 for a subset of our most vulnerable patients (termed difference-in-differences; Stata SE 16.0).

**Results:**

In our study of Waves 1 and 2, we offered vaccinations to 24,410 patients. Of those, 6,712 (27.5%) scheduled the vaccine at BILH (Table 1). Patients in Wave 1 were much more likely to be vaccinated at BILH than patients in Wave 2. Patients offered the vaccine in Wave 1 and living in Dorchester were 1.7 percentage points more likely to be vaccinated at all (p=0.445) and 9.4 percentage points more likely to be vaccinated at BILH than another site in MA (p-value = 0.001), relative to patients living outside of Dorchester and offered the vaccine in Wave 2 (Table 2).

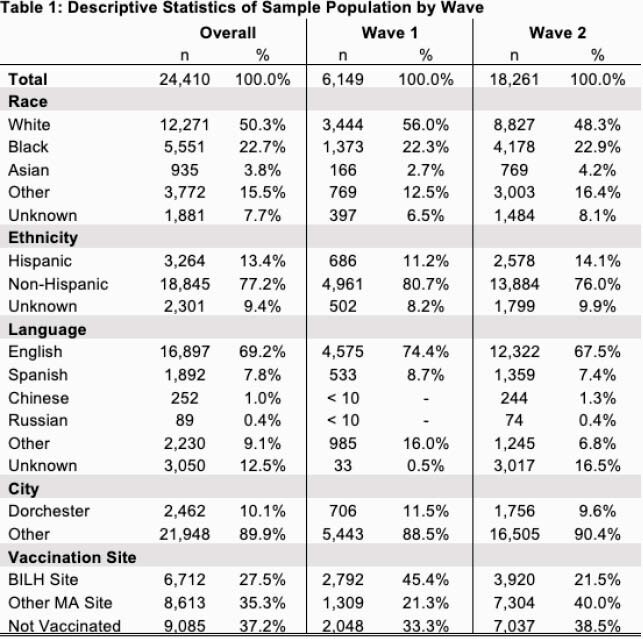

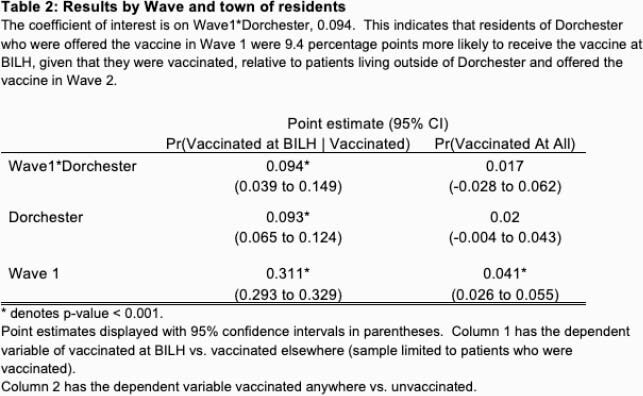

The coefficient of interest is on Wave1*Dorchester, 0.094. This indicates that residents of Dorchester who were offered the vaccine in Wave 1 were 9.4 percentage points more likely to receive the vaccine at BILH, given that they were vaccinated, relative to patients living outside of Dorchester and offered the vaccine in Wave 2.

**Conclusion:**

Patients residing in an urban community given prioritized access to vaccination had a higher likelihood of vaccination at our health system, given that they were vaccinated, than patients in other urban communities without prioritized access. We provide an example of a successful effort to move towards equity in access to COVID-19- vaccines, in contrast to larger national trends.^2,3^ Health systems can use a prioritization approach to improve vaccination equity.

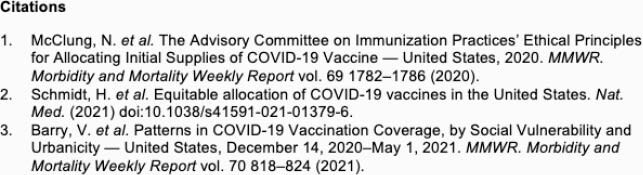

**Disclosures:**

**All Authors**: No reported disclosures

